# Effects of Oral Nutritional Supplement with β-Hydroxy-β-methylbutyrate (HMB) on Biochemical and Hematological Indices in Community-Dwelling Older Adults at Risk of Malnutrition: Findings from the SHIELD Study

**DOI:** 10.3390/nu16152495

**Published:** 2024-07-31

**Authors:** Siew Ling Tey, Dieu Thi Thu Huynh, Sing Teang Kong, Jeffery Oliver, Geraldine Baggs, Yen Ling Low, Choon How How, Magdalin Cheong, Wai Leng Chow, Ngiap Chuan Tan, Tar Choon Aw, Samuel Teong Huang Chew

**Affiliations:** 1 Abbott Nutrition Research and Development, Singapore 138668, Singapore; dieu.huynh@abbott.com (D.T.T.H.); singteang.kong@abbott.com (S.T.K.); yenling.low@abbott.com (Y.L.L.); 2 Abbott Nutrition Research and Development, Columbus, OH 43219, USA; jeffery.oliver@abbott.com (J.O.); geraldine.baggs@abbott.com (G.B.); 3 Care and Health Integration, Changi General Hospital, Singapore 529889, Singapore; how.choon.how@singhealth.com.sg; 4 Department of Dietetic & Food Services, Changi General Hospital, Singapore 529889, Singapore; magdalin.cheong@singhealth.com.sg; 5 Health Services Research, Changi General Hospital, Singapore 529889, Singapore; chow_wai_leng@moh.gov.sg; 6 SingHealth Polyclinics, Singapore 150167, Singapore; tan.ngiap.chuan@singhealth.com.sg; 7 Department of Laboratory Medicine, Changi General Hospital, Singapore 529889, Singapore; aw.tar.choon@singhealth.com.sg; 8 Duke-NUS Graduate School of Medicine, 8 College Road, Singapore 169857, Singapore; samuel.chew.t.h@singhealth.com.sg; 9 Yong Loo Lin School of Medicine, National University of Singapore, 10 Medical Drive, Singapore 117597, Singapore; 10 Department of Geriatric Medicine, Changi General Hospital, Singapore 529889, Singapore

**Keywords:** aging, older adults, malnutrition, biochemical indices, hematological indices, oral nutritional supplement, β-hydroxy-β-methylbutyrate (HMB)

## Abstract

Malnutrition may result in abnormal biochemical and hematological indices. This planned prespecified analysis investigated the effects of a specialized oral nutritional supplement (ONS) on biochemical and hematological indices in community-dwelling older adults at risk of malnutrition. In the Strengthening Health in ELDerly through nutrition (SHIELD) study, 811 older adults aged 65 years and above took part in this randomized, double-blind, placebo-controlled, multi-center study. Participants were randomly allocated to either a complete and balanced specialized ONS (each serving provides 262 kcal, 10.5 g protein, 7.75 µg vitamin D_3_, and 0.74 g calcium β-hydroxy-β-methylbutyrate) and dietary counselling (intervention group) or a placebo and dietary counselling (placebo group). Both groups consumed study products twice a day for 180 days. Data were collected at baseline, day 90, and day 180. Blood analysis results at follow-up visits were analyzed using repeated measures analysis of covariance with adjustments for confounders. Overall, when compared with the placebo group, the intervention group showed significantly greater urea (6.0 mmol/L vs. 5.4 mmol/L, *p* < 0.001), urea to creatinine ratio (4.39 vs. 4.26, *p* < 0.001), prealbumin (24.9 mg/dL vs. 24.0 mg/dL, *p* < 0.001), vitamin B_12_ (480.0 pmol/L vs. 420.1 pmol/L, *p* < 0.001), and globulin levels (26.8 g/L vs. 26.5 g/L, *p* = 0.032). The intervention group also had a significantly higher absolute reticulocyte count (62.0 × 10^3^/µL vs. 58.2 × 10^3^/µL, overall *p* < 0.001) and mean platelet volume (10.0 fL vs. 9.9 fL, overall *p* = 0.003). Furthermore, significant improvements were seen in total protein at day 90 (71.7 g/L vs. 71.1 g/L, *p* = 0.017) and in absolute monocyte count at day 90 (0.50 × 10^3^/µL vs. 0.47 × 10^3^/µL, *p* = 0.009) in the intervention group. In conclusion, daily consumption of a specialized ONS for six months led to significant improvements in biochemical and hematological indices in community-dwelling older adults at risk of malnutrition.

## 1. Introduction

The concept of healthy aging is drawing considerable attention as the world population of older people expands. The number of people older than 60 years is expected to increase from 1 billion in 2019 to more than double or 2.1 billion by 2050 [1,2]. Predictions suggest that at least half of these older adults will be living in Asia, as population growth of older adults is projected to rise exponentially in Asia while leveling off in Europe and North America [3]. For healthy aging, older people want to *age well*, which they describe as maintaining physical and mental health and sustaining their sense of well-being, i.e., qualities key to independent living [4].

On average, one in three community-dwelling older adults are at risk of malnutrition [5,6,7,8,9,10,11,12,13] as a result of social isolation, anorexia of aging, and health issues [14,15,16,17]. Poor nutrition compromises healthy aging because of its association with poor muscle health, limited ability to recover from acute illness or injury, and ultimately, functional decline and mortality [18,19,20]. Muscle loss is further worsened by aging-associated vulnerability to acute critical illness, cancer, neurological disorders, chronic inflammatory conditions, diabetes, and disease-related malnutrition [19,21,22]. Various interventional strategies have been proposed to counteract aging-related muscle loss, especially nutrition and progressive resistance exercise training [23,24,25]. A targeted nutritional approach is essential for older adults with malnutrition or its risk to ensure that they meet their daily requirements for energy and protein [26,27]. A comprehensive review published in *New England Journal of Medicine* in 2024 highlighted the underlying pathophysiological pathway of malnutrition and the evidence-based interventions to address malnutrition [28]. Specific recommendations calling for more adherence to nutritional guidelines, and the process of care for older adults across the continuum of care was emphasized in a recent review by Dent at al. [29].

When older adults with or at risk of malnutrition cannot meet nutritional requirements by consumption of food alone, oral nutritional supplements (ONSs) are often advised to provide energy, protein, and other nutrients [27,28,29]. Results of randomized controlled trials (RCTs) [30,31,32], as well as systematic reviews and meta-analyses of RCTs [33,34,35], have evidenced a range of ONS-related nutritional, functional, and clinical benefits in adult populations consisting of mainly older people. Based on older adults with aging-related anorexia, a systematic review and meta-analysis showed positive effects on overall appetite, energy intake, body weight, and body mass index (BMI) [36]. Another recent systematic review and meta-analysis by Cawood and colleagues reported that poorly nourished older people in hospital and community settings experienced fewer complications (infections, pressure ulcers, wound and fracture healing) when they consumed ONS daily over multi-month intervals (mean 74 days) [35]. Multi-nutrient ONS interventions (in comparison with placebo) led to a significant improvement in physical performance measures (chair rise time and handgrip strength) in study participants with frailty or sarcopenia or in those affected by specific medical conditions [37].

Two systematic reviews and meta-analyses on β-hydroxy-β-methylbutyrate (HMB) reported increased muscle strength in adult patients and preservation of muscle strength and function in older adults with frailty and sarcopenia [38,39]. Underlying mechanisms for HMB include stimulation of muscle protein synthesis and down-regulation of proteolysis [40]. Interventions using ONS with appropriate proportions of macronutrients and a wide range of essential micronutrients together with HMB have been shown to improve quality of life, nutritional outcomes, and function in both community-dwelling and hospitalized older populations, especially those with sarcopenia, prefrailty, or frailty [31,41,42,43,44,45]. From here forth, ONS containing HMB is defined as specialized ONS in this paper. Results from our prior report on community-living older adults in Singapore (Strengthening Health in ELDerly through nutrition, SHIELD) show benefits of 6-month daily consumption of specialized ONS, including improved nutritional and functional outcomes—nutritional intake and status, body weight, mid-upper arm circumference, serum 25-hydroxyvitamin D levels, and handgrip and leg strength, compared to placebo [30].

Although nutritional and functional outcomes are important, our current study draws attention to biochemical and hematological indices that may be useful to predict disease prognosis and mortality outcomes in adults with acute and chronic diseases, including conditions associated with malnutrition. In a systematic review by Zhang et al., researchers noted that concentrations of serum albumin, hemoglobin, prealbumin, and total protein were significantly lower in people with malnutrition risk than for those without risk [46]. Similarly, Keller reported on other biochemical markers related to nutritional status—prealbumin, albumin, transferrin, retinol binding protein, urinary creatinine, zinc, and vitamins A, B_1_, B_6_, B_12_, and folate; of these, prealbumin and albumin appeared to be readily measured laboratory values useful for prediction of surgical outcomes, with low prealbumin and albumin levels associated with increased mortality in people with severe illness and with overall mortality in older adults [47].

A recent study consisting of three patient cohorts with malnutrition, cancer, and neurological diseases reported that the use of medical nutrition therapy resulted in higher levels of erythrocytes and albumin and lower levels of C-reactive protein at six months and twelve months in patients with malnutrition [48]. Further, Pereira et al. found increases in immunoglobulins, myoglobin, total protein, vitamin E, and magnesium following 12-week intervention with ONS enriched with protein, vitamin D, and HMB; inflammation-related ferritin and osteopontin decreased, suggesting decreased inflammation [49]. In an RCT, Peng et al. showed a significant increase in serum vitamin D levels in the intervention group (ONS with HMB), compared to the control group [45]. However, a majority of these studies were limited by small sample sizes [45,48,49], heterogenous study populations [48], and the small number of different blood biomarkers that were analyzed [45,48].

Therefore, the SHIELD study aimed to address the knowledge gaps described above and to investigate the effects of a 6-month nutritional intervention (specialized ONS) on a wide range of biochemical and hematological indices in community-dwelling older adults with malnutrition risk.

## 2. Materials and Methods

### 2.1. Study Design

The SHIELD study involved community-dwelling older people aged 65 years and older in Singapore. The study participants were recruited between August 2017 and September 2019, and the last participant completed the six-month intervention in March 2020.

This study was conducted in accordance with the ethical principles that have their origins in the Declaration of Helsinki. The study was approved by the SingHealth Centralized Institutional Review Board in Singapore (reference number 2017/2273, approval date 23 May 2017). All enrolled participants provided written informed consent. The study was registered at clinicaltrials.gov as NCT03240952.

The full description of the study had been previously published [30]. This paper reports the results from the planned prespecified analysis of biochemical and hematological outcomes of the SHIELD study.

In brief, this was a randomized, double-blind, placebo-controlled, multi-center study. Participants were randomly allocated to one of the two treatments: specialized ONS or placebo. The specialized ONS provided complete and balanced nutrition; each serving contained 262 kcal, 10.5 g protein, 8.5 g fat, 34.2 g carbohydrate, 7.75 µg (310 IU) vitamin D_3_, and 0.74 g calcium HMB (Ensure, Abbott Nutrition, Singapore). Each serving of the placebo supplement contained 60 kcal, 1.07 g protein, 1.21 g fat, and 11.9 g carbohydrate. Both study products were milk-based, vanilla in flavor, and packaged in identical sachets for masking purpose. In addition, dietary counselling was provided to both groups. Participants were asked to consume the study product as a supplement twice a day for 180 days.

Randomization schedules were computer generated using a pseudo-random permuted blocks algorithm. An electronic data capture system was used to assign participant numbers and randomized participants to study product codes based on the generated randomization schedules. This study was double-blind in that the investigators, study staff, and participants were not aware of the identity of the study products. Laboratory personnel were also blinded throughout the study. Study products were delivered and collected by a healthcare services provider who was independent of the study.

### 2.2. Study Participants

Study participants were recruited from the general public, community centers, senior activity centers, polyclinics, and hospitals in Singapore. Participants were eligible for inclusion in the study if they met the following criteria: male or female aged ≥ 65 years, community-dwelling, and ambulant with or without aid. Participants had to be at medium or high risk of malnutrition based on the Malnutrition Universal Screening Tool (MUST) [50], which was used to identify malnutrition risk in community-dwelling older adults. MUST consists of three components: BMI, weight loss, and acute disease that can affect risk of malnutrition. BMI and weight loss components each have a score of 0 to 2, and acute disease has a score of 2 only. Participants were assessed for all three components, and the sum of their scores classified them into one of three categories of malnutrition risk: low risk (score = 0), medium risk (score = 1), and high risk (score ≥ 2) [50].

Individuals with stable medical conditions (defined as long-term medical conditions treated with regular medication such that symptoms were what was expected by the participant when well) were included in the study. Individuals were excluded if they had allergies or intolerance to milk products, dementia, diabetes, active infectious disease, severe gastrointestinal disorder, cystic fibrosis, end-stage organ failure, pre-terminal disease, acute myocardial infarction in the last 30 days, or active malignancy in the last five years.

### 2.3. Study Procedures

Socio-demographic information, including age and sex, co-morbidities, and malnutrition status using MUST, were collected at baseline. Modified Barthel Index was used to assess activities of daily living [51], and Charlson Comorbidity Index score was used to determine the severity and number of comorbidities [52]. Physical activity level was assessed using Physical Activity Scale for the Elderly (PASE) [53,54].

Body weight and composition were measured using Tanita MC-780 at baseline, day 90, and day 180. Blood samples were also collected at baseline, day 90, and day 180 via venipuncture for the analyses of biochemical and hematological indices, of which 68% (544 out of 805) of the participants provided fasting blood samples and participation for fasting blood sampling was voluntary. Serum CRP and prealbumin levels were determined using COBAS c502; sodium, potassium, chloride, urea, creatinine, corrected calcium, glucose, total bilirubin, alkaline phosphatase, alanine transaminase, aspartate transaminase, total protein, albumin, and globulin were analyzed using COBAS c702; ferritin, 25(OH)D, and vitamin B12 were analyzed using COBAS e801; the full blood count was analyzed using Sysmex XN9000. Fasting blood samples were used to analyze zinc levels (Inductively Coupled Plasma Mass Spectrometry). Estimated Glomerular Filtration Rate (eGFR) was calculated using CKD-EPI equation [55].

In this study, adherence to study products was calculated over 180 days using the return of unopened sachets and intake records of the participants. The compliance percentage was calculated using the following formula: number of sachets consumed divided by number of sachets required to consume, multiplied by 100.

### 2.4. Data Analysis

This manuscript reports the planned prespecified analysis results of the SHIELD study on biochemical and hematological indices. Baseline characteristics of the study participants were reported as means and standard error for continuous variables and as numbers and percentages for categorical variables. For continuous variables, normality of the data was assessed using the Shapiro–Wilk test (*p* < 0.001) and graphical methods. Blood analysis results at follow-up visits were analyzed using repeated measures analysis of covariance with factors for visit, study group, baseline age, baseline BMI, hospital discharge in the last 30 days, baseline MUST risk, sex, study group by visit, study group by sex, and baseline value.

All the analyses were conducted using the modified intent to treat (MITT) dataset. MITT was defined as all available data from all participants who received at least one study feeding. SAS version 9.4 (SAS Institute, Cary, NC, USA) was used for all statistical analyses. *p* < 0.05 was considered statistically significant.

## 3. Results

### 3.1. Baseline Characteristics

A total of 3094 individuals were screened for eligibility; 1567 were excluded as they failed to fulfil the inclusion criteria, and a further 716 declined to take part in this study [30]. Thus, 811 community-dwelling older adults at risk of malnutrition aged 65 years and older were eligible for the SHIELD study. Of these, 406 received specialized ONS and dietary counselling, and 405 received placebo and dietary counselling. Six participants did not receive their allocated intervention (five from intervention and one from placebo) and were excluded from primary analysis.

In terms of sex, approximately 40% of the participants were male and 60% were female. All study participants were at risk of malnutrition as determined by MUST, with 52.2% classified as medium risk and 47.8% as high risk. The mean body weight of the study participants at baseline was 45.3 kg, BMI was 18.4 kg/m^2^, and fat mass was 8.1 kg. The mean age was 74.15 years old, and 93% had a Charlson Comorbidity Index score of 0, reflecting a relatively healthy cohort. Approximately 89% had a modified Barthel Index score of 100 out of 100, and the mean PASE score was 104.1.

Measures for biochemical and hematological indices at baseline are shown in Table 1. The mean levels of all the biochemical and hematological indices were within the normal ranges. Most participants had biochemical and hematological indices values that were within the normal ranges (Table S1). Baseline biochemical and hematological indices by sex are shown in Table S2.

### 3.2. Product Compliance

Product compliance over 180 days was relatively high in both groups, 72% in the intervention and 81% in the placebo group [30].

### 3.3. Body Weight and Composition, Physical Activity Level

Both intervention and placebo groups had increases in body weight, BMI, and fat mass over 180 days, with a significantly higher increase in the intervention group compared to the placebo (all *p* < 0.001). At day 180, the intervention group had significantly higher body weight (47.3 kg vs. 46.2 kg), BMI (19.2 kg/m^2^ vs. 18.8 kg/m^2^), and fat mass (9.5 kg vs. 8.4 kg) compared with the placebo group [30].

There was no statistically significant difference in PASE score over 180 days between the intervention group and the placebo group (*p* = 0.889).

### 3.4. Biochemical Indices

In the total cohort, levels of potassium, urea, urea to creatinine ratio, prealbumin, and vitamin B_12_ were significantly greater in the intervention group compared to the placebo group over 180 days, as well as at day 90 and day 180 (all *p* ≤ 0.010) (Table 2). Corrected calcium and globulin levels were also significantly higher in the intervention group over 180 days and at day 90 (all *p* ≤ 0.032), as was the total protein level at day 90 (*p* = 0.017) in the intervention group compared to the placebo group (Table 2). There were no statistically significant differences in blood glucose and eGFR levels between the groups (both overall *p* ≥ 0.116).

Similar results were found when results were analyzed by sex; the intervention group had overall significantly higher urea, urea– to creatinine ratio, prealbumin, and vitamin B_12_ levels than the placebo group in both males (all overall *p* ≤ 0.008; Table S3a) and females (all overall *p* ≤ 0.012) (Table S3b). In the female cohort, corrected calcium, globulin levels (both overall *p* ≤ 0.040), and total protein at day 90 were significantly higher in the intervention group (*p* = 0.022; Table S3b).

### 3.5. Hematological Indices

As shown in Table 3, mean platelet volume (MPV), absolute reticulocyte count, and reticulocyte percentage were significantly greater, while the lymphocyte percentage was lower in the intervention group compared to the placebo group over 180 days (all overall *p* ≤ 0.044) in the total cohort. The intervention group had significantly higher levels for hematocrit, absolute monocyte count, and monocyte percentage than the placebo at day 90 (all *p* ≤ 0.045).

At day 90, significant improvements in absolute reticulocyte count and reticulocyte percentage were also found in both male and female cohorts (all overall *p* ≤ 0.008; Table S4a,b). These improvements at day 90 were sustained through day 180 (all *p* ≤ 0.024; Table S4a,b).

## 4. Discussion

The results of our present analysis of SHIELD study data show that daily consumption of specialized ONS for six months led to significant improvements in biochemical and hematological indices. Due to the nature of the study design, the improvements are attributed to the intervention product as a whole rather than to any single ingredient or nutrient. Values for biochemical parameters, such as urea, prealbumin, vitamin B_12_, and globulin, were significantly greater in the intervention group compared to the placebo group over the 6-month time course. Hematological parameter values were likewise improved, i.e., mean platelet volume, absolute reticulocyte count, and reticulocyte percentage were significantly higher in the specialized ONS group. In addition, we observed significant improvements in total protein and in absolute monocyte count and monocyte percentage at day 90, which we attributed to consumption of specialized ONS. Such findings complement our earlier findings of improvements in nutritional and functional outcomes, as evidenced by better nutritional intake and status and beneficial increases in body weight, mid upper-arm circumference, serum 25-hydroxyvitamin D levels, and handgrip and leg strength [30].

### 4.1. Biochemical Indices

#### 4.1.1. Prealbumin

Prealbumin is a common surrogate marker used to investigate nutritional status in patients who are clinically stable. It is often used as a marker for malnutrition and has a shorter half-life (2–3 days) compared to albumin; hence, it may be more useful as an indicator of recent poor nutritional intake [47,56,57]. It is also a negative acute phase reactant, so its levels are suppressed in the presence of inflammation [47,57,58]. Therefore, this effect needs to be taken into consideration when interpreting results in patients who are acutely ill or are in a chronic inflammatory state.

Our present study in older adults with malnutrition risk is one of the first to demonstrate significant increases in prealbumin after 90 days of nutritional intervention, and increases that were sustained until day 180, compared to the placebo group. While 80% of participants had prealbumin levels within the normal range at the start, those in the intervention group showed significant increases in prealbumin levels at day 90 and beyond, i.e., an indication of improved nutritional status. However, other ONS studies in older adults showed mixed findings, with some showing improvement in prealbumin in populations with sarcopenia or high prevalence of sarcopenia [49,59], while others showing no significant difference in hospitalized, recently discharged, and pre-frail populations [45,60,61] between intervention and control groups. These contradictory results are most likely due to the heterogeneity in the study cohorts. For example, in hospitalized and recently discharged populations [60,61], it is likely that the lack of significant differences may be related to recovery from acute illness and resolution of inflammation. Similarly, in the pre-frail population study, the participants were not malnourished or at risk of malnutrition based on the average score from the Mini Nutritional Assessment Short-Form (MNA-SF) [45], unlike the population included in this study, where all the participants were at medium or high risk of malnutrition [30] and 76% had evidence of sarcopenia [62] using the Asian Working Group for Sarcopenia 2019 criteria [63]. It is also possible that the use of an ONS with high protein and HMB in the two positive studies may have led to a higher level of prealbumin in the intervention group [49,59].

#### 4.1.2. Urea and Urea to Creatinine Ratio

The urea and urea to creatinine ratio were also significantly higher in the intervention group as compared to the placebo group. A similar finding of higher urea in the ONS group was observed in an earlier study [64], while no changes were found in other studies [65,66,67]. In these studies reporting no significant differences, urine nitrogen was measured instead of blood urea [67], sample size was small [67], and total protein intake per day was not reported [64,65,66]; it is difficult to relate these findings to the results of our present study.

Urea is produced in the liver from breakdown of protein and amino acid, and it is often used as an indicator of protein intake [68]. On the other hand, creatinine is a by-product of the metabolism of creatine, of which 95% is found in skeletal muscles, and is hence a surrogate marker of muscle mass [69]. Serum creatinine is lower in women due to lower muscle mass [56], thus contributing to the higher urea to creatinine ratio in females. Urea to creatinine ratio was demonstrated to have a linear relationship with protein intake and could be a useful estimate of dietary protein intake [70]. Higher urea and urea to creatinine ratio have been associated with higher protein intake; conversely, low urea and urea to creatinine ratio indicate lower protein intake in people with normal renal function [68]. This is an important finding, as the National Nutrition Survey 2022 in Singapore reported that one in two adults aged 50 to 69 years did not meet their dietary protein requirement [71]. Hence, an easily accessible clinical index that can identify low protein intake may be helpful in identifying individuals who need further nutritional assessment and intervention.

#### 4.1.3. C-Reactive Protein

C-reactive protein level was reversed from being higher in the intervention group at baseline (intervention 5.15 mg/L vs. placebo 3.84 mg/L) to lower than the placebo group at the end of the study (intervention 4.77 mg/L vs. placebo 5.69 mg/L), although this change was not statistically significant. CRP is raised in inflammation [72] and aging; hence, it is an important indicator of inflammaging.

The relationship between nutrition and inflammation is complex [73] but important, as reduced food intake was associated with increased CRP levels [74]. In the secondary analysis of the EFFORT trial, nutritional intervention in hospitalized adults at risk of malnutrition reduced mortality only in the cohorts with low to medium CRP levels (<100 mg/L) at baseline [75]. This observation was followed by a recent study, which reported a four-fold reduction in CRP after 12 months of medical nutrition therapy (baseline: 20.13 mg/L vs. 12 months: 4.86 mg/L) in patients with malnutrition [48]. These studies suggest that long-term nutritional therapy may help reduce inflammation and improve clinical outcomes, particularly in people who are malnourished with elevated levels of CRP (<100 mg/L) at baseline.

#### 4.1.4. Vitamin B_12_

The present study showed a significant increase in vitamin B_12_ levels at day 90 and day 180. This increase was likely due to the fact that two servings of the specialized ONS provide 1.82 µg of vitamin B_12_, which is 76% of the Recommended Dietary Allowance for vitamin B_12_ in adults aged 51 and above [76]. Vitamin B_12_ is essential for proper functioning of nerve cells and cellular metabolism, and deficiency can lead to neuropathy and macrocytic anemia, which are both reversible [77,78].

#### 4.1.5. Globulin

Total protein includes albumin and globulin. Globulins are made up of immunoglobulins, protein carriers, enzymes, and complement factors [79]. In general, increase in globulin can be triggered by infections, liver disease, and connective tissue disease and malignancies, while its decrease can be caused by malnutrition or nephrotic syndrome [79,80]. In the present study, we found that total protein and globulin levels were higher in the intervention group compared to the placebo group. Given that albumin levels remained stable in this study, the increase in total protein was most likely driven by an increase in globulin. In line with this finding, a recent study reported that treatment with ONS enriched with protein, vitamin D, and HMB significantly increased immunoglobulin, sex hormone-binding globulin, transferrin, and complement C3 levels as compared to the baseline in participants who were at risk or had malnutrition [49], which is likely a result of better overall nutrition.

### 4.2. Hematological Indices

#### 4.2.1. Reticulocytes

Immature red blood cells (RBCs) are made in bone marrow and released into bloodstream as reticulocytes [81]. Reticulocytes continue to mature in the spleen or bloodstream to form RBCs. In normal conditions, most red cells exist as mature RBCs, with only a very small proportion of reticulocytes. Absolute reticulocyte count and reticulocyte percentage are rarely measured in clinical studies of ONS use. In the present study, the significantly higher absolute reticulocyte count and reticulocyte percentage could be indicative of heightened erythropoiesis [81,82], as a result of better nutrition with ONS intervention. The intervention group showed a decrease in the proportion of participants with low absolute reticulocyte count (7.7% baseline vs. 4.7% at day 180) as compared to the placebo group (7.4% at baseline vs. 7.3% at day 180). A previous study reported that induced malnutrition in young rats was associated with a reduction in reticulocyte percentage, which was reversible by improving nutrition [83]. The increase in reticulocytes was accompanied by a significant increase in hematocrit at day 90 in the present study, suggesting that nutrition improved hematopoiesis.

#### 4.2.2. Monocytes

Among the leukocytes, monocytes are the largest in size and third largest in quantity [84]. Despite being present in small quantities, monocytes play an important role in fighting infection and clearance of foreign material from the body [84]. In this study, absolute monocyte count was significantly increased in males at day 90. A previous study in male athletes also reported increases in blood monocyte count after 6 weeks of HMB supplementation compared to controls [85]. This increase in monocyte count in poorly nourished older adults who received nutritional intervention offers an interesting insight; additional studies are needed to investigate this relationship further.

#### 4.2.3. Mean Platelet Volume

Platelets are cells that help blood clotting. Mean platelet volume is the measure of the average platelet size [84] and is not commonly reported in ONS studies. When there is an increase in platelet production or destruction, newly generated platelet size gets larger as a result of increased thrombopoiesis [86,87]. We found a significant increase in mean platelet volume in the intervention group, which was driven by an increase in female participants, suggesting an increase in platelet production in females with ONS supplementation. This would be in keeping with our findings on reticulocytes and monocytes in the intervention group.

### 4.3. Strengths and Limitations

The strengths of this study include the large sample size and a wide range of biochemical and hematological indices measured over a period of six months. The improvements observed cannot be attributed to a single ingredient or nutrient but only to the specialized ONS as a whole. Although the results are statistically significant, the absolute changes observed require further studies to ascertain the clinical significance of these improvements, as many of the levels are still within the normal range. As our study consists of free-living older adults, it is possible that we were not able to account for all potential confounding factors. Despite this potential limitation, we found positive results in the intervention group, suggesting that the results would likely be more significant if repeated in a controlled feeding study environment. In addition, the results of this study are based on relatively healthy community-dwelling older adults with malnutrition risk, which may affect the generalizability of the study findings. Further research with a similar intervention and study design is required in older patients who are overtly malnourished and have abnormal biochemical and hematological indices to examine whether the positive results of our study can be extended to a more severely malnourished cohort.

## 5. Conclusions

To our knowledge, this is the first study to investigate the effects of an ONS containing HMB on a wide range of biochemical and hematological indices in a large cohort of community-dwelling older adults at risk of malnutrition.

In this study of older adults with malnutrition risk, we found that daily consumption of a specialized ONS for six months significantly improved biochemical and hematological indices. Such nutritional intervention led to significant increases in urea, prealbumin, vitamin B_12_, and globulin levels compared to the placebo, along with improvement of hematological measures of reticulocytes and monocytes (absolute number and its percentage) and mean platelet volume.

We thus recommend early nutritional intervention as an effective way to achieve better nutritional status and to improve biochemical and hematological indices in community-dwelling older adults at risk of malnutrition.

## Figures and Tables

**Table 1:** Biochemical and hematological indices in the intervention and placebo groups at baseline.

	Total	Intervention	Placebo	*p*-Value
	(*n* = 805)	(*n* = 401)	(*n* = 404)	
**Biochemical indices**				
Sodium (mmol/L)	141.2 ± 0.1	141.1 ± 0.2	141.3 ± 0.2	0.308
Potassium (mmol/L)	4.5 ± 0.02	4.5 ± 0.02	4.5 ± 0.02	0.957
Chloride (mmol/L)	101.8 ± 0.1	101.7 ± 0.2	101.8 ± 0.2	0.527
Urea (mmol/L)	5.2 ± 0.1	5.2 ± 0.1	5.1 ± 0.1	0.678
Creatinine (µmol/L)	73.7 ± 0.8	73.7 ± 1.0	73.6 ± 1.1	0.970
Urea to creatinine ratio ^	4.25 ± 0.01	4.25 ± 0.01	4.24 ± 0.01	0.632
Glucose (mmol/L)	5.4 ± 0.03	5.4 ± 0.04	5.4 ± 0.05	0.703
eGFR (mL/min/1.73 m^2^)	78.0 ± 0.6	78.1 ± 0.8	78.0 ± 0.8	0.937
CRP (mg/L)	4.5 ± 0.4	5.2 ± 0.8	3.8 ± 0.4	0.130
	(*n* = 470)	(*n* = 227)	(*n* = 243)	
Ferritin (µg/L)	234.7 ± 9.9	255.3 ± 18.0	214.3 ± 8.2	**0.038**
Prealbumin (mg/dL)	23.8 ± 0.2	23.7 ± 0.2	23.9 ± 0.2	0.470
Corrected calcium (mmol/L)	2.23 ± 0.003	2.22 ± 0.010	2.23 ± 0.004	0.232
Vitamin B_12_ (pmol/L)	469.7 ± 8.3	460.1 ± 11.8	479.3 ± 11.8	0.250
	(*n* = 790)	(*n* = 395)	(*n* = 395)	
Zinc (µg/L)	817.0 ± 4.7	820.2 ± 7.0	813.9 ± 6.2	0.498
	(*n* = 544)	(*n* = 272)	(*n* = 272)	
Total bilirubin (µmol/L)	10.9 ± 0.4	11.5 ± 0.7	10.3 ± 0.2	0.114
	(*n* = 803)	(*n* = 399)		
ALP (U/L)	71.1 ± 1.0	72.4 ± 1.7	69.9 ± 1.1	0.232
ALT (U/L)	18.4 ± 0.5	18.4 ± 0.8	18.4 ± 0.5	0.920
	(*n* = 804)		(*n* = 403)	
AST (U/L)	24.8 ± 0.5	25.1 ± 0.9	24.4 ± 0.4	0.420
Total protein (g/L)	71.5 ± 0.2	71.5 ± 0.2	71.5 ± 0.2	0.972
Albumin (g/L)	45.2 ± 0.1	45.1 ± 0.1	45.3 ± 0.2	0.524
Globulin (g/L)	26.4 ± 0.2	26.5 ± 0.2	26.3 ± 0.2	0.672
**Hematological indices**				
Hemoglobin (g/dL)	13.1 ± 0.1	13.1 ± 0.1	13.0 ± 0.1	0.141
Hematocrit (%)	39.7 ± 0.1	39.9 ± 0.2	39.5 ± 0.2	0.233
MCV (fL)	90.3 ± 0.3	90.5 ± 0.4	90.1 ± 0.4	0.446
MCH (pg)	29.7 ± 0.1	29.8 ± 0.2	29.6 ± 0.1	0.312
MCHC (g/dL)	32.9 ± 0.04	32.9 ± 0.05	32.8 ± 0.05	0.243
RDW (%)	13.49 ± 0.05	13.42 ± 0.07	13.55 ± 0.08	0.184
Platelet count (10^3^/µL)	231.6 ± 2.3	230.0 ± 3.2	233.1 ± 3.3	0.504
MPV (fL)	9.9 ± 0.03	9.9 ± 0.04	9.9 ± 0.04	0.686
	(*n* = 793)	(*n* = 394)	(*n* = 399)	
WBC count (10^3^/µL)	5.6 ± 0.1	5.5 ± 0.1	5.7 ± 0.1	0.176
Neutrophils (absolute) (10^3^/µL)	3.4 ± 0.1	3.3 ± 0.1	3.4 ± 0.1	0.161
Lymphocytes (absolute) (10^3^/µL)	1.6 ± 0.02	1.6 ± 0.03	1.6 ± 0.03	0.556
Monocytes (absolute) (10^3^/µL)	0.46 ± 0.01	0.45 ± 0.01	0.47 ± 0.01	0.187
Eosinophils (absolute) (10^3^/µL)	0.20 ± 0.01	0.20 ± 0.01	0.19 ± 0.01	0.434
Basophils (absolute) (10^3^/µL)	0.04 ± 0.002	0.04 ± 0.003	0.04 ± 0.003	0.258
Neutrophils (%)	58.6 ± 0.3	58.5 ± 0.5	58.7 ± 0.5	0.739
Lymphocytes (%)	29.1 ± 0.3	29.1 ± 0.4	29.1 ± 0.4	0.928
Monocytes (%)	8.2 ± 0.1	8.1 ± 0.1	8.2 ± 0.1	0.590
Eosinophils (%)	3.3 ± 0.1	3.4 ± 0.2	3.2 ± 0.1	0.412
Basophils (%)	0.80 ± 0.01	0.84 ± 0.02	0.77 ± 0.02	**0.020**
RBC count (10^6^/µL)	4.4 ± 0.02	4.4 ± 0.03	4.4 ± 0.03	0.678
Reticulocytes (absolute) (10^3^/µL)	56.1 ± 0.6	57.0 ± 0.9	55.2 ± 0.8	0.129
Reticulocytes (%)	1.3 ± 0.01	1.3 ± 0.02	1.3 ± 0.02	0.301

ALT, alanine transaminase. ALP, alkaline phosphatase. AST, aspartate transaminase. CRP, C-reactive protein. eGFR, estimated glomerular filtration rate. MCH, mean corpuscular hemoglobin. MCHC, mean corpuscular hemoglobin concentration. MCV, mean corpuscular volume. MPV, mean platelet volume. RBC, red blood cell. RDW, red cell distribution width. WBC, white blood cell. All values are presented as mean ± SEM. SEM: Standard error of the mean. *p*-value is from analysis of variance with treatment group as a factor. ^ Urea to creatinine ratio was log-transformed due to skewed residuals. When the sample sizes are less than the overall stated sample sizes, the actual sample sizes are specified. The bolded values are statistically significant (*p* < 0.05).

**Table 2:** Biochemical indices at day 90 and day 180 in the intervention and placebo groups.

Biochemical Indices	Overall			Day 90			Day 180		
	Intervention	Placebo	*p*-Value	Intervention	Placebo	*p*-Value	Intervention	Placebo	*p*-Value
	(*n* = 627)	(*n* = 610)		(*n* = 317)	(*n* = 313)		(*n* = 310)	(*n* = 297)	
Sodium (mmol/L)	140.8 ± 0.1	141.0 ± 0.1	0.253	140.8 ± 0.2	140.9 ± 0.2	0.647	140.9 ± 0.2	141.2 ± 0.2	0.139
Potassium (mmol/L)	4.6 ± 0.02	4.5 ± 0.02	**0.001**	4.6 ± 0.03	4.5 ± 0.03	**0.004**	4.6 ± 0.03	4.5 ± 0.03	**0.010**
	(*n* = 626)	(*n* = 609)		(*n* = 316)				(*n* = 296)	
Chloride (mmol/L)	101.7 ± 0.2	102.0 ± 0.2	**0.036**	101.5 ± 0.2	101.8 ± 0.2	0.055	101.9 ± 0.2	102.2 ± 0.2	0.095
Urea (mmol/L)	6.0 ± 0.1	5.4 ± 0.1	**<0.001**	6.1 ± 0.1	5.4 ± 0.1	**<0.001**	5.9 ± 0.1	5.4 ± 0.1	**<0.001**
Creatinine (µmol/L)	73.3 ± 0.7	74.2 ± 0.7	0.198	73.7 ± 0.7	74.5 ± 0.7	0.241	72.9 ± 0.7	73.8 ± 0.8	0.279
Urea– to creatinine ratio ^	4.39 ± 0.01	4.26 ± 0.02	**<0.001**	4.40 ± 0.02	4.25 ± 0.02	**<0.001**	4.37 ± 0.02	4.26 ± 0.02	**<0.001**
Glucose (mmol/L)	5.5 ± 0.1	5.4 ± 0.1	0.116	5.4 ± 0.1	5.3 ± 0.1	0.167	5.5 ± 0.1	5.4 ± 0.1	0.162
eGFR (mL/min/1.73 m^2^)	78.7 ± 0.5	78.2 ± 0.5	0.367	78.4 ± 0.6	77.8 ± 0.6	0.354	79.0 ± 0.6	78.6 ± 0.6	0.511
CRP (mg/L)	5.3 ± 0.8	5.7 ± 0.8	0.627	5.8 ± 1.0	5.7 ± 1.0	0.933	4.8 ± 0.9	5.7 ± 0.9	0.313
	(*n* = 286)	(*n* = 277)		(*n* = 143)	(*n* = 146)		(*n* = 143)	(*n* = 131)	
Ferritin (µg/L)	200.1 ± 5.0	204.8 ± 5.0	0.327	196.7 ± 5.2	205.4 ± 5.2	0.104	203.4 ± 5.3	204.2 ± 5.4	0.884
Prealbumin * (mg/dL)	24.9 ± 0.2	24.0 ± 0.2	**<0.001**	25.2 ± 0.2	24.1 ± 0.2	**<0.001**	24.5 ± 0.2	23.8 ± 0.2	**0.003**
Corrected calcium (mmol/L)	2.24 ± 0.004	2.23 ± 0.005	**0.022**	2.24 ± 0.005	2.23 ± 0.005	**0.026**	2.24 ± 0.005	2.23 ± 0.005	0.139
Vitamin B_12_ (pmol/L)	480.0 ± 8.9	420.1 ± 9.0	**<0.001**	471.6 ± 8.9	418.9 ± 9.0	**<0.001**	488.5 ± 9.7	421.2 ± 9.8	**<0.001**
	(*n* = 615)	(*n* = 596)		(*n* = 312)	(*n* = 304)		(*n* = 303)	(*n* = 292)	
Zinc (µg/L)	819.5 ± 9.8	825.0 ± 9.8	0.469	821.5 ± 10.4	824.3 ± 10.5	0.759	817.4 ± 10.6	825.8 ± 10.4	0.370
	(*n* =397)	(*n* = 410)		(*n* = 202)	(*n* = 204)		(*n* = 195)	(*n* = 206)	
Total bilirubin (µmol/L)	10.8 ± 0.2	11.0 ± 0.2	0.334	10.7 ± 0.3	10.8 ± 0.3	0.697	10.9 ± 0.3	11.3 ± 0.3	0.199
	(*n* = 624)			(*n* = 315)			(*n* = 309)		
ALP (U/L)	63.9 ± 0.9	64.8 ± 0.9	0.290	64.2 ± 0.9	64.8 ± 0.9	0.535	63.5 ± 1.0	64.8 ± 1.0	0.240
ALT (U/L)	18.0 ± 0.8	16.5 ± 0.8	0.074	18.3 ± 0.7	17.0 ± 0.7	0.059	17.8 ± 1.1	16.0 ± 1.1	0.225
		(*n* = 609)						(*n* = 296)	
AST (U/L)	24.1 ± 0.7	23.0 ± 0.7	0.125	24.3 ± 0.7	23.5 ± 0.7	0.231	24.0 ± 0.9	22.6 ± 0.9	0.200
Total protein (g/L)	71.7 ± 0.2	71.3 ± 0.2	0.075	71.7 ± 0.2	71.1 ± 0.2	**0.017**	71.8 ± 0.3	71.6 ± 0.3	0.450
Albumin * (g/L)	44.8 ± 0.1	44.8 ± 0.1	0.879	44.8 ± 0.2	44.9 ± 0.2	0.600	44.8 ± 0.2	44.8 ± 0.2	0.811
Globulin (g/L)	26.8 ± 0.2	26.5 ± 0.2	**0.032**	26.8 ± 0.2	26.2 ± 0.2	**0.004**	26.9 ± 0.2	26.8 ± 0.2	0.374

ALT, alanine transaminase. ALP, alkaline phosphatase. AST, aspartate transaminase. CRP, C-reactive protein. eGFR, estimated glomerular filtration rate. All values are presented as LSM ± SE. LSM: least squares mean, SE: standard error. LSMs are from repeated measures analysis of covariance with factors for visit, study group, study group by visit interaction, sex, study group by sex interaction, study group by sex by visit interaction, hospital admission in the last 30 days at baseline, baseline MUST risk, baseline age, baseline BMI, and baseline measurement. The three-way interaction was dropped from the model if it was not significant. D90 *p* and D180 *p* are the unadjusted *p*-values from the study group comparisons at D90 and D180 from the study group by visit interaction. * Results from model which included significant three-way interaction study group by sex by visit interaction. ^ Urea– to creatinine ratio was log-transformed due to skewed residuals. When the sample sizes are less than the overall stated sample sizes, the actual sample sizes are specified. The bolded values are statistically significant (*p* < 0.05).

**Table 3:** Hematological indices at day 90 and day 180 in the intervention and placebo groups.

Hematological Indices	Overall			Day 90			Day 180		
	Intervention	Placebo	*p*-Value	Intervention	Placebo	*p*-Value	Intervention	Placebo	*p*-Value
	(*n* = 624)	(*n* = 611)		(*n* = 316)	(*n* = 314)		(*n* = 308)	(*n* = 297)	
Hemoglobin (g/dL)	13.2 ± 0.05	13.1 ± 0.05	0.149	13.3 ± 0.1	13.2 ± 0.1	0.064	13.2 ± 0.1	13.1 ± 0.1	0.428
Hematocrit (%)	39.9 ± 0.2	39.7 ± 0.2	0.143	40.1 ± 0.2	39.7 ± 0.2	**0.045**	39.7 ± 0.2	39.6 ± 0.2	0.483
MCV * (fL)	90.3 ± 0.2	90.0 ± 0.2	0.071	90.2 ± 0.2	89.9 ± 0.2	0.123	90.3 ± 0.2	90.0 ± 0.2	0.077
MCH * (pg)	29.9 ± 0.1	29.9 ± 0.1	0.268	29.9 ± 0.1	29.9 ± 0.1	0.504	30.0 ± 0.1	29.9 ± 0.1	0.200
MCHC (g/dL)	33.2 ± 0.1	33.2 ± 0.1	0.950	33.2 ± 0.1	33.2 ± 0.1	0.716	33.2 ± 0.1	33.2 ± 0.1	0.845
RDW (%)	13.25 ± 0.05	13.28 ± 0.05	0.504	13.24 ± 0.05	13.25 ± 0.05	0.855	13.26 ± 0.05	13.31 ± 0.05	0.326
Platelet count (10^3^/µL)	213.9 ± 2.9	217.6 ± 2.9	0.184	213.9 ± 3.0	217.7 ± 3.0	0.210	213.9 ± 3.1	217.5 ± 3.1	0.262
MPV (fL)	10.0 ± 0.03	9.9 ± 0.03	**0.003**	10.1 ± 0.04	9.9 ± 0.04	**<0.001**	10.0 ± 0.04	9.9 ± 0.04	0.080
	(*n* = 610)	(*n* = 603)		(*n* = 309)	(*n* = 310)		(*n* = 301)	(*n* = 293)	
WBC count (10^3^/µL)	5.8 ± 0.1	5.7 ± 0.1	0.750	5.8 ± 0.1	5.8 ± 0.1	0.482	5.7 ± 0.1	5.7 ± 0.1	0.779
		(*n* = 610)			(*n* = 313)				
Neutrophils (absolute) (10^3^/µL)	3.4 ± 0.1	3.4 ± 0.1	0.750	3.5 ± 0.1	3.4 ± 0.1	0.520	3.3 ± 0.1	3.4 ± 0.1	0.797
Lymphocytes (absolute) (10^3^/µL)	1.6 ± 0.03	1.7 ± 0.03	0.319	1.6 ± 0.03	1.7 ± 0.03	0.267	1.7 ± 0.03	1.7 ± 0.03	0.518
Monocytes (absolute) * (10^3^/µL)	0.49 ± 0.01	0.47 ± 0.01	0.095	0.50 ± 0.01	0.47 ± 0.01	**0.009**	0.48 ± 0.01	0.48 ± 0.01	0.855
Eosinophils (absolute) (10^3^/µL)	0.21 ± 0.01	0.20 ± 0.01	0.110	0.22 ± 0.01	0.20 ± 0.01	0.141	0.21 ± 0.01	0.19 ± 0.01	0.205
Basophils (absolute) (10^3^/µL)	0.04 ± 0.003	0.04 ± 0.003	0.715	0.04 ± 0.003	0.04 ± 0.003	0.631	0.04 ± 0.003	0.04 ± 0.003	0.894
Neutrophils (%)	58.1 ± 0.5	57.8 ± 0.5	0.435	58.4 ± 0.5	57.8 ± 0.5	0.258	57.8 ± 0.5	57.7 ± 0.5	0.831
Lymphocytes (%)	28.9 ± 0.4	29.7 ± 0.4	**0.044**	28.6 ± 0.5	29.7 ± 0.5	**0.015**	29.2 ±0.4	29.7 ± 0.5	0.294
Monocytes (%)	8.6 ± 0.1	8.4 ± 0.1	0.061	8.6 ± 0.1	8.4 ± 0.1	**0.033**	8.6 ± 0.1	8.5 ± 0.1	0.242
Eosinophils * (%)	3.5 ± 0.1	3.3 ± 0.1	0.101	3.5 ± 0.2	3.3 ± 0.2	0.328	3.5 ± 0.1	3.2 ± 0.2	0.059
Basophils (%)	0.77 ± 0.02	0.78 ± 0.02	0.872	0.78 ± 0.02	0.77 ± 0.02	0.692	0.77 ± 0.02	0.78 ± 0.02	0.474
RBC count (10^6^/µL)	4.5 ± 0.02	4.5 ± 0.02	0.579	4.5 ± 0.02	4.5 ± 0.02	0.190	4.4 ± 0.02	4.4 ± 0.02	0.862
Reticulocytes (absolute) (10^3^/µL)	62.0 ± 0.8	58.2 ± 0.9	**<0.001**	61.5 ± 0.9	57.3 ± 0.9	**<0.001**	62.6 ± 0.9	59.1 ± 0.9	**<0.001**
Reticulocytes (%)	1.4 ± 0.02	1.3 ± 0.02	**<0.001**	1.4 ± 0.02	1.3 ± 0.02	**<0.001**	1.4 ± 0.02	1.3 ± 0.02	**0.001**

MCH, mean corpuscular hemoglobin. MCHC, mean corpuscular hemoglobin concentration. MCV, mean corpuscular volume. MPV, mean platelet volume. RBC, red blood cell. RDW, red cell distribution width. WBC, white blood cell. All values are presented as LSM ± SE, unless otherwise stated. LSM: least squares mean, SE: standard error. LSMs are from repeated measures analysis of covariance with factors for visit, study group, study group by visit interaction, sex, study group by sex interaction, study group by sex by visit interaction, hospital admission in the last 30 days at baseline, baseline MUST risk, baseline age, baseline BMI, and baseline measurement. The three-way interaction was dropped from the model if it was not significant. D90 *p* and D180 *p* are the unadjusted *p*-values from the study group comparisons at D90 and D180 from the study group by visit interaction. * Results from model which included significant three-way interaction study group by sex by visit interaction. When the sample sizes are less than the overall stated sample sizes, the actual sample sizes are specified. The bolded values are statistically significant (*p* < 0.05).

## Data Availability

The original contributions presented in the study are included in the article/Supplementary Material; further inquiries can be directed to the corresponding author.

## Supplementary Materials

The following supporting information can be downloaded at https://www.mdpi.com/article/10.3390/nu16152495/s1. Table S1. Baseline biochemical and hematological category by intervention; Table S2. Biochemical and hematological indices in the intervention and placebo groups at baseline by sex; Table S3. Biochemical indices at day 90 and day 180 for males and females; Table S4. Hematological indices at day 90 and day 180 for males and females.
