# Supplementary material for: Effects of Oral Nutritional Supplement with β-Hydroxy-β-methylbutyrate (HMB) on Biochemical and Hematological Indices in Community-Dwelling Older Adults at Risk of Malnutrition: Findings from the SHIELD Study
Source: Nutrients. 2024 Jul 31;16(15):2495. doi: 10.3390/nu16152495 (PMC11313967; doi:10.3390/nu16152495)
Supplement: Supplementary file 1 [file nutrients-16-02495-s001.zip › nutrients-3085411-supplementary.pdf]

Table S1: Baseline biochemical and hematological category by intervention

Indices	Status	Reference range	Total *n* (%)	Intervention *n* (%)	Placebo *n* (%)
Biochemical indices					
Sodium (mmol/L)	Low	<135.0	26 (3.2)	14 (3.5)	12 (3.0)
	Normal	135.0 – 145.0	748 (92.9)	374 (93.3)	374 (92.6)
	High	>145.0	31 (3.9)	13 (3.2)	18 (4.5)
Potassium (mmol/L)	Low	<3.5	2 (0.2)	2 (0.5)	0 (0.0)
	Normal	3.5 – 5.3	772 (95.9)	385 (96.0)	387 (95.8)
	High	>5.3	31 (3.9)	14 (3.5)	17 (4.2)
Chloride (mmol/L)	Low	<96	27 (3.4)	11 (2.7)	16 (4.0)
	Normal	96 – 108	773 (96.0)	389 (97.0)	384 (95.0)
	High	>108	5 (0.6)	1 (0.2)	4 (1.0)
Urea (mmol/L)	Low	<2.8	11 (1.4)	2 (0.5)	9 (2.2)
	Normal	2.8 – 7.7	743 (92.3)	375 (93.5)	368 (91.1)
	High	>7.7	51 (6.3)	24 (6.0)	27 (6.7)
Creatinine (µmol/L)					
Men	Low	<65	29 (9.1)	20 (11.7)	9 (6.0)
	Normal	65 – 125	273 (85.3)	142 (83.0)	131 (87.9)
	High	>125	18 (5.6)	9 (5.3)	9 (6.0)
Women	Low	<50	60 (12.4)	25 (10.9)	35 (13.7)
	Normal	50 – 90	396 (81.6)	191 (83.0)	205 (80.4)
	High	>90	29 (6.0)	14 (6.1)	15 (5.9)
Glucose (mmol/L)	Normal	3.1 – 5.6	453 (83.1)	229 (84.2)	224 (82.1)
	High	5.7 - 6.9	86 (15.8)	39 (14.3)	47 (17.2)
	Very high	>6.9	6 (1.1)	4 (1.5)	2 (0.7)
eGFR (mL/min/1.73m^2)	Normal or high	≥90	189 (23.5)	93 (23.2)	96 (23.8)
	Mildly decreased	60 - 89	498 (61.9)	253 (63.1)	245 (60.6)
	Moderately decreased	30 - 59	114 (14.2)	54 (13.5)	60 (14.9)
	Severely decreased	15-29	4 (0.5)	1 (0.2)	3 (0.7)
CRP (mg/L)	Low	<1.0	146 (31.1)	70 (30.8)	76 (31.3)
	Normal	1.0 – 3.0	203 (43.2)	99 (43.6)	104 (42.8)
	High	>3.0	121 (25.7)	58 (25.6)	63 (25.9)
Ferritin (µg/L)					
Men	Low	<32	11 (3.4)	5 (2.9)	6 (4.0)
	Normal	32-294	216 (67.5)	114 (66.7)	102 (68.5)

	High	>294	93 (29.1)	52 (30.4)	41 (27.5)
Women	Low	<18.2	4 (0.8)	1 (0.4)	3 (1.2)
	Normal	18.2-339	419 (86.4)	197 (85.7)	222 (87.1
	High	>339	62 (12.8)	32 (13.9)	30 (11.8)
Prealbumin (mg/dL)	Low	<20	143 (17.8)	74 (18.5)	69 (17.1)
	Normal	20 – 40	662 (82.2)	327 (81.5)	335 (82.9)
	High	>40	0 (0.0)	0 (0.0)	0 (0.0)
Corrected calcium (mmol/L)	Low	<2.10	3 (0.4)	0 (0.0)	3 (0.7)
	Normal	2.10 – 2.60	793 (98.5)	398 (99.3)	395 (97.8)
	High	>2.60	9 (1.1)	3 (0.7)	6 (1.5)
Vitamin B_{12} (pmol/L)	Low	<132	1 (0.1)	0 (0.0)	1 (0.3)
	Normal	132 – 835	729 (92.3)	368 (93.2)	361 (91.4)
	High	>835	60 (7.6)	27 (6.8)	33 (8.4)
Zinc (µg/L)	Low	<724	91 (16.7)	50 (18.4)	41 (15.1)
	Normal	724 - 1244	452 (83.1)	221 (81.3)	231 (84.9)
	High	>1244	1 (0.2)	1 (0.4)	0 (0.0)
Total bilirubin (µmol/L)	Low	<5.0	48 (6.0)	26 (6.5)	22 (5.4)
	Normal	5.0 – 30.0	750 (93.4)	368 (92.2)	382 (94.6)
	High	>30.0	5 (0.6)	5 (1.3)	0 (0.0)
ALP (U/L)	Low	<32.0	4 (0.5)	2 (0.5)	2 (0.5)
	Normal	32.0 – 103.0	742 (92.2)	368 (91.8)	374 (92.6)
	High	>103.0	59 (7.3)	31 (7.7)	28 (6.9)
ALT (U/L)	Low	<10	49 (6.1)	28 (7.0)	21 (5.2)
	Normal	10.0 – 55.0	749 (93.2)	369 (92.0)	380 (94.3)
	High	>55.0	6 (0.7)	4 (1.0)	2 (0.5)
AST (U/L)	Low	<10.0	0 (0.0)	0 (0.0)	0 (0.0)
	Normal	10.0 – 45.0	787 (97.8)	390 (97.3)	397 (98.3)
	High	>45.0	18 (2.2)	11 (2.7)	7 (1.7)
Total protein (g/L)	Low	<62	7 (0.9)	3 (0.7)	4 (1.0)
	Normal	62 – 82	789 (98.0)	390 (97.3)	399 (98.8)
	High	>82	9 (1.1)	8 (2.0)	1 (0.2)
Albumin (g/L)	Low	<37	9 (1.1)	3 (0.7)	6 (1.5)
	Normal	37 – 51	789 (98.0)	396 (98.8)	393 (97.3)
	High	>51	7 (0.9)	2 (0.5)	5 (1.2)
Globulin (g/L)	Low	<20	18 (2.2)	5 (1.2)	13 (3.2)
	Normal	20 - 36	767 (95.3)	385 (96.0)	382 (94.6)
	High	>36	20 (2.5)	11 (2.7)	9 (2.2)
Hematological indices					

Hemoglobin (g/dL)					
Men	Low	<13.0	28 (8.8)	12 (7.0)	16 (10.7)
	Normal	13.0 – 17.0	244 (76.2)	131 (76.6)	113 (75.8)
	High	>17.0	48 (15.00)	28 (16.4)	20 (13.4)
Women	Low	<11.5	61 (12.6)	26 (11.3)	35 (13.7)
	Normal	11.5 – 15.0	417 (86.0)	200 (87.0)	217 (85.1)
	High	>15.0	7 (1.4)	4 (1.7)	3 (1.2)
Hematocrit (%)					
Men	Low	<38.0	39 (12.2)	17 (9.9)	22 (14.8)
	Normal	38.0 – 52.0	247 (77.2)	137 (80.1)	110 (73.8)
	High	>52.0	34 (10.6)	17 (9.9)	17 (11.4)
Women	Low	<36.0	74 (15.3)	32 (13.9)	42 (16.5)
	Normal	36.0 – 46.0	404 (83.3)	196 (85.2)	208 (81.6)
	High	>46.0	7 (1.4)	2 (0.9)	5 (2.0)
MCV (fL)	Low	<76.0	55 (6.8)	27 (6.7)	28 (6.9)
	Normal	76.0 – 96.0	634 (78.8)	311 (77.6)	323 (80.0)
	High	>96.0	116 (14.4)	63 (15.7)	53 (13.1)
MCH (pg)	Low	<27.0	82 (10.2)	39 (9.7)	43 (10.6)
	Normal	27.0 – 32.0	616 (76.5)	302 (75.3)	314 (77.7)
	High	>32.0	107 (13.3)	60 (15.0)	47 (11.6)
MCHC (g/dL)	Low	<32.0	122 (15.2)	61 (15.2)	61 (15.1)
	Normal	32.0 – 36.0	680 (84.5)	338 (84.3)	342 (84.7)
	High	>36.0	3 (0.4)	2 (0.5)	1 (0.2)
RDW (%)	Low	<10.90	0 (0.0)	0 (0.0)	0 (0.0)
	Normal	10.90 – 15.70	744 (92.4)	372 (92.8)	372 (92.1)
	High	>15.70	61 (7.6)	29 (7.2)	32 (7.9)
Platelet count (10^3/µL)	Low	<150	51 (6.3)	25 (6.2)	26 (6.4)
	Normal	150 – 450	745 (92.5)	371 (92.5)	374 (92.6)
	High	>450	9 (1.1)	5 (1.2)	4 (1.0)
MPV (fL)	Low	<6.3	0 (0.0)	0 (0.0)	0 (0.0)
	Normal	6.3 - 10.1	507 (63.9)	254 (64.5)	253 (63.4)
	High	>10.1	286 (36.1)	140 (35.5)	146 (36.6)
WBC count (10^3/µL)	Low	<4.0	101 (12.5)	58 (14.5)	43 (10.6)
	Normal	4.0 - 10.0	692 (86.0)	340 (84.8)	352 (87.1)
	High	>10.0	12 (1.5)	3 (0.7)	9 (2.2)
Neutrophils (absolute) (10^3/µL)	Low	<2.0	75 (9.3)	48 (12.0)	27 (6.7)
	Normal	2.0 – 7.5	720 (89.4)	351 (87.5)	369 (91.3)
	High	>7.5	10 (1.2)	2 (0.5)	8 (2.0)

Lymphocytes (absolute) ($10^3/\mu L$)	Low	<1.0	76 (9.4)	41 (10.2)	35 (8.7)
	Normal	1.0 – 3.0	716 (88.9)	351 (87.5)	365 (90.3)
	High	>3.0	13 (1.6)	9 (2.2)	4 (1.0)
Monocytes (absolute) ($10^3/\mu L$)	Low	<0.2	4 (0.5)	1 (0.2)	3 (0.7)
	Normal	0.2 – 0.8	785 (97.5)	393 (98.0)	392 (97.0)
	High	>0.8	16 (2.0)	7 (1.7)	9 (2.2)
Eosinophils (absolute) ($10^3/\mu L$)	Low	<0.04	79 (9.8)	46 (11.5)	33 (8.2)
	Normal	0.04 – 0.44	668 (83.0)	325 (81.0)	343 (84.9)
	High	>0.44	58 (7.2)	30 (7.5)	28 (6.9)
Basophils (absolute) ($10^3/\mu L$)	Normal	0.0 – 0.1	800 (99.4)	400 (99.8)	400 (99.0)
	High	>0.1	5 (0.6)	1 (0.2)	4 (1.0)
Neutrophils (%)	Low	<40.0	23 (2.9)	10 (2.5)	13 (3.2)
	Normal	40.0 – 75.0	746 (92.7)	373 (93.0)	373 (92.3)
	High	>75.0	36 (4.5)	18 (4.5)	18 (4.5)
Lymphocytes (%)	Low	<15.0	38 (4.7)	19 (4.7)	19 (4.7)
	Normal	15.0 – 41.0	697 (86.6)	343 (85.5)	354 (87.6)
	High	>41.0	70 (8.7)	39 (9.7)	31 (7.7)
Monocytes (%)	Low	<2.0	0 (0.0)	0 (0.0)	0 (0.0)
	Normal	2.0 – 10.0	674 (83.7)	337 (84.0)	337 (83.4)
	High	>10.0	131 (16.3)	64 (16.0)	67 (16.6)
Eosinophils (%)	Normal	0.0 – 6.0	723 (89.8)	359 (89.5)	364 (90.1)
	High	>6.0	82 (10.2)	42 (10.5)	40 (9.9)
Basophils (%)	Normal	0.0 – 1.0	626 (77.8)	300 (74.8)	326 (80.7)
	High	>1.0	179 (22.2)	101 (25.2)	78 (19.3)
RBC count ($10^6/\mu L$)					
Men	Low	<4.3	40 (12.5)	21 (12.3)	19 (12.8)
	Normal	4.3 – 6.3	261 (81.6)	145 (84.8)	116 (77.9)
	High	>6.3	19 (5.9)	5 (2.9)	14 (9.4)
Women	Low	<4.0	86 (17.7)	37 (16.1)	49 (19.2)
	Normal	4.0 – 5.5	384 (79.2)	183 (79.6)	201 (78.8)
	High	>5.5	15 (3.1)	10 (4.3)	5 (2.0)
Reticulocytes (absolute) ($10^3/\mu L$)	Low	<36.0	15 (1.9)	31 (7.7)	30 (7.4)
	Normal	36.0 – 126.0	786 (97.6)	368 (91.8)	374 (92.6)
	High	>126.0	4 (0.5)	2 (0.5)	0 (0.0)
Reticulocytes (count) (%)	Low	<0.7	61 (7.6)	4 (1.0)	11 (2.7)
	Normal	0.7 – 2.7	742 (92.2)	394 (98.3)	392 (97.0)
	High	>2.7	2 (0.2)	3 (0.7)	1 (0.2)

Table S2: Biochemical and hematological indices in the intervention and placebo groups at baseline by sex

	Male		*p*-Value	Female		*p*-Value
	**Intervention** (*n* = 171)	**Placebo** (*n* = 149)		**Intervention** (*n* = 230)	**Placebo** (*n* = 255)	
Biochemical indices						
Sodium (mmol/L)	140.4 ± 0.3	140.5 ± 0.3	0.610	141.6 ± 0.2	141.8 ± 0.2	0.637
Potassium (mmol/L)	4.5 ± 0.04	4.6 ± 0.04	0.464	4.5 ± 0.03	4.5 ± 0.03	0.726
Chloride (mmol/L)	101.2 ± 0.3	101.3 ± 0.3	0.831	102.1 ± 0.2	102.2 ± 0.2	0.742
Urea (mmol/L)	5.3 ± 0.1	5.5 ± 0.2	0.509	5.1 ± 0.1	5.0 ± 0.1	0.406
Creatinine (µmol/L)	86.1 ± 1.5	90.5 ± 1.9	**0.031**	64.5 ± 1.0	63.8 ± 1.0	0.682
Urea to creatinine ratio ^	4.11 ± 0.02	4.08 ± 0.02	0.179	4.35 ± 0.02	4.34 ± 0.02	0.531
Glucose (mmol/L)	5.5 ± 0.1	5.5 ± 0.1	0.777	5.3 ± 0.1	5.4 ± 0.1	0.672
eGFR (mL/min/1.73m^2)	76.2 ± 1.2	73.1 ± 1.3	0.066	79.4 ± 1.0	80.8 ± 0.9	0.311
CRP (mg/L)	6.4 ± 1.2 (*n* = 103)	4.6 ± 0.9 (*n* = 90)	0.194	4.1 ± 1.0 (*n* = 124)	3.4 ± 0.5 (*n* = 153)	0.507
Ferritin (µg/L)	276.7 ± 25.9	244.6 ± 13.9	0.306	239.4 ± 24.8	196.7 ± 10.0	0.094
Prealbumin (mg/dL)	24.1 ± 0.4	25.0 ± 0.4	0.094	23.3 ± 0.3	23.3 ± 0.3	0.887
Corrected calcium (mmol/L)	2.21 ± 0.01	2.22 ± 0.01	0.092	2.24 ± 0.01	2.24 ± 0.01	0.941
Vitamin B_{12} (pmol/L)	420.1 ± 16.8 (*n* = 170)	425.6 ± 16.1 (*n* = 145)	0.834	490.4 ± 16.1 (*n* = 225)	510.4 ± 15.7 (*n* = 250)	0.345
Zinc (µg/L)	805.7 ± 11.7 (*n* = 113)	803.5 ± 11.7 (*n* = 91)	0.886	830.5 ± 8.4 (*n* = 159)	819.1 ± 7.2 (*n* = 181)	0.333
Total bilirubin (µmol/L)	13.7 ± 1.6 (*n* = 170)	11.0 ± 0.4	**0.022**	9.8 ± 0.3 (*n* = 229)	9.9 ± 0.2	0.962
ALP (U/L)	75.6 ± 3.5	68.3 ± 1.6	**0.026**	70.0 ± 1.4	70.9 ± 1.5	0.746
ALT (U/L)	17.6 ± 0.8	19.1 ± 0.8	0.293	18.9 ± 1.14	18.0 ± 0.7 (*n* = 254)	0.464
AST (U/L)	23.6 ± 1.0	24.0 ± 0.6	0.776	26.3 ± 1.3	24.6 ± 0.5	0.159
Total protein (g/L)	71.5 ± 0.4	70.9 ± 0.4	0.221	71.6 ± 0.3	71.9 ± 0.3	0.370
Albumin (g/L)	44.8 ± 0.2	44.6 ± 0.3	0.588	45.4 ± 0.2	45.7 ± 0.2	0.339
Globulin (g/L)	26.7 ± 0.4	26.3 ± 0.4	0.354	26.3 ± 0.3	26.4 ± 0.3	0.796
Hematological indices						
Hemoglobin (g/dL)	13.6 ± 0.1	13.4 ± 0.1	0.137	12.8 ± 0.1	12.7 ± 0.1	0.876
Hematocrit (%)	41.1 ± 0.3	40.7 ± 0.4	0.338	39.0 ± 0.2	38.9 ± 0.2	0.787
MCV (fL)	91.1 ± 0.6	89.1 ± 0.8	**0.020**	90.1 ± 0.5	90.7 ± 0.4	0.384
MCH (pg)	30.2 ± 0.2	29.4 ± 0.3	**0.012**	29.5 ± 0.2	29.7 ± 0.1	0.417
MCHC (g/dL)	33.2 ± 0.1	33.0 ± 0.1	0.073	32.7 ± 0.1	32.8 ± 0.1	0.796
RDW (%)	13.51 ± 0.11	13.89 ± 0.13	**0.017**	13.35 ± 0.08	13.36 ± 0.09	0.976
Platelet count (10^3/µL)	225.0 ± 4.9	235.1 ± 6.2	0.167	233.8 ± 4.3	232.0 ± 3.8	0.757
MPV (fL)	9.8 ± 0.1	9.8 ± 0.1	0.881	10.0 ± 0.1	10.0 ± 0.1	0.967

	(n = 168)	(n = 144)		(n = 226)		
WBC count (10^3/μL)	5.8 ± 0.1	6.2 ± 0.0	0.054	5.3 ± 0.1	5.4 ± 0.1	0.567
Neutrophils (absolute) (10^3/μL)	3.5 ± 0.1	3.8 ± 0.2	0.091	3.1 ± 0.1	3.2 ± 0.1	0.449
Lymphocytes (absolute) (10^3/μL)	1.6 ± 0.04	1.6 ± 0.04	0.365	1.6 ± 0.04	1.6 ± 0.03	0.993
Monocytes (absolute) (10^3/μL)	0.48 ± 0.01	0.54 ± 0.02	**0.007**	0.42 ± 0.01	0.42 ± 0.01	0.948
Eosinophils (absolute) (10^3/μL)	0.28 ± 0.02	0.28 ± 0.03	0.976	0.15 ± 0.01	0.14 ± 0.01	0.669
Basophils (absolute) (10^3/μL)	0.05 ± 0.004	0.05 ± 0.004	0.866	0.04 ± 0.003	0.03 ± 0.003	0.163
Neutrophils (%)	59.1 ± 0.7	59.2 ± 0.8	0.925	58.0 ± 0.7	58.4 ± 0.6	0.649
Lymphocytes (%)	27.2 ± 0.6	27.0 ± 0.7	0.813	30.5 ± 0.6	30.3 ± 0.5	0.742
Monocytes (%)	8.4 ± 0.2	8.7 ± 0.3	0.287	7.9 ± 0.1	7.9 ± 0.1	0.881
Eosinophils (%)	4.4 ± 0.3	4.3 ± 0.3	0.863	2.7 ± 0.2	2.6 ± 0.1	0.699
Basophils (%)	0.84 ± 0.03	0.77 ± 0.03	0.094	0.83 ± 0.03	0.78 ± 0.02	0.101
RBC count (10^6/μL)	4.5 ± 0.04	4.6 ± 0.06	0.242	4.4 ± 0.04	4.3 ± 0.03	0.295
Reticulocytes (absolute) (10^3/μL)	55.3 ± 1.3	53.0 ± 1.3	0.222	58.2 ± 1.2	56.5 ± 1.0	0.241
Reticulocytes (count) (%)	1.2 ± 0.03	1.2 ± 0.03	0.116	1.4 ± 0.03	1.3 ± 0.03	0.639

ALT, alanine transaminase. ALP, alkaline phosphatase. AST, aspartate transaminase. CRP, C-reactive protein. eGFR, estimated glomerular filtration rate. MCH, mean corpuscular hemoglobin. MCHC, mean corpuscular hemoglobin concentration. MCV, mean corpuscular volume. MPV, mean platelet volume. RBC, red blood cell. RDW, red cell distribution width. WBC, white blood cell. All values are presented as Mean ± SEM. SEM: Standard Error of the Mean, The statistical model contains study group, sex, and study group by sex interaction. The *p*-values are from the difference of Least Squares Means between study groups for each sex. ^ Urea to creatinine ratio was log-transformed due to skewed residuals. When the sample sizes are less than the overall stated sample sizes, the actual sample sizes are specified. The bolded values are statistically significant ($p < 0.05$).

Table S3a: Biochemical indices at day 90 and day 180 for males

Biochemical indices	Overall			Day 90			Day 180		
	Intervention	Placebo	*p*-Value	Intervention	Placebo	*p*-Value	Intervention	Placebo	*p*-Value
	(*n* = 259)	(*n* = 212)		(*n* = 133)	(*n* = 109)		(*n* = 126)	(*n* = 103)	
Sodium (mmol/L)	140.6 ± 0.2	140.8 ± 0.2	0.454	140.5 ± 0.2	140.6 ± 0.2	0.658	140.8 ± 0.2	141.0 ± 0.2	0.421
Potassium (mmol/L)	4.7 ± 0.03	4.6 ± 0.03	**0.007**	4.7 ± 0.04	4.6 ± 0.04	**0.013**	4.6 ± 0.04	4.5 ± 0.04	0.060
		(*n* = 211)						(*n* = 102)	
Chloride (mmol/L)	101.6 ± 0.2	101.8 ± 0.2	0.394	101.3 ± 0.2	101.6 ± 0.2	0.367	102.0 ± 0.2	102.1 ± 0.2	0.587
Urea (mmol/L)	6.0 ± 0.1	5.5 ± 0.1	**<0.001**	6.2 ± 0.1	5.3 ± 0.1	**<0.001**	5.9 ± 0.1	5.6 ± 0.2	0.101
Creatinine (µmol/L)	74.5 ± 0.9	75.5 ± 0.9	0.363	74.6 ± 0.9	75.6 ± 1.0	0.333	74.5 ± 1.0	75.4 ± 1.1	0.520
Urea to creatinine ratio ^	4.32 ± 0.02	4.20 ± 0.02	**<0.001**	4.34 ± 0.02	4.18 ± 0.02	**<0.001**	4.29 ± 0.02	4.22 ± 0.02	**0.010**
Glucose (mmol/L)	5.4 ± 0.1	5.4 ± 0.1	0.462	5.5 ± 0.1	5.3 ± 0.1	0.234	5.4 ± 0.1	5.4 ± 0.1	0.840
eGFR (mL/min/1.73m^2)	78.0 ± 0.7	77.7 ± 0.7	0.727	78.1 ± 0.7	77.2 ± 0.8	0.316	77.8 ± 0.7	78.2 ± 0.8	0.703
CRP (mg/L)	6.2 ± 1.1	7.2 ± 1.1	0.473	7.3 ± 1.4	6.78 ± 1.5	0.782	5.1 ± 1.2	7.6 ± 1.2	0.093
	(*n* = 119)	(*n* = 96)		(*n* = 61)	(*n* = 50)		(*n* = 58)	(*n* = 46)	
Ferritin (µg/L)	208.5 ± 6.3	209.2 ± 6.5	0.931	208.4 ± 6.7	208.9 ± 7.0	0.951	208.6 ± 6.9	209.4 ± 7.3	0.928
Prealbumin (mg/dL)	25.1 ± 0.3	24.0 ± 0.3	**0.002**	25.4 ± 0.3	24.4 ± 0.3	**0.012**	24.8 ± 0.3	23.6 ± 0.3	**0.004**
Corrected calcium (mmol/L)	2.24 ± 0.01	2.23 ± 0.01	0.189	2.24 ± 0.01	2.23 ± 0.01	0.099	2.24 ± 0.01	2.24 ± 0.01	0.665
Vitamin B_{12} (pmol/L)	456.1 ± 11.3	419.4 ± 11.8	**0.008**	453.0 ± 11.3	419.4 ± 11.8	**0.015**	459.3 ± 12.8	419.5 ± 13.5	**0.015**
	(*n* = 256)	(*n* = 209)		(*n* = 132)	(*n* = 107)		(*n* = 124)	(*n* = 102)	
Zinc (µg/L)	804.6 ± 11.7	828.7 ± 12.2	0.050	807.0 ± 12.7	827.2 ± 13.7	0.169	802.2 ± 13.3	830.3 ± 13.7	0.064
	(*n* = 159)	(*n* = 127)		(*n* = 82)	(*n* = 61)		(*n* = 77)	(*n* = 66)	
Total bilirubin (µmol/L)	11.1 ± 0.3	11.2 ± 0.3	0.781	10.9 ± 0.3	11.1 ± 0.4	0.648	11.4 ± 0.34	11.4 ± 0.4	0.981
ALP (U/L)	64.6 ± 1.1	64.7 ± 1.2	0.914	64.8 ± 1.1	64.6 ± 1.2	0.882	64.4 ± 1.4	64.9 ± 1.4	0.777
ALT (U/L)	17.9 ± 1.0	17.1 ± 1.1	0.547	18.7 ± 0.9	17.5 ± 0.9	0.250	17.1 ± 1.6	16.6 ± 1.8	0.859
		(*n* = 211)						(*n* = 102)	
AST (U/L)	23.7 ± 0.9	23.0 ± 0.9	0.544	24.2 ± 0.8	23.6 ± 0.9	0.542	23.2 ± 1.2	22.5 ± 1.3	0.676
Total protein (g/L)	71.6 ± 0.3	71.2 ± 0.3	0.316	71.6 ± 0.3	71.2 ± 0.3	0.288	71.6 ± 0.3	71.3 ± 0.4	0.506
Albumin (g/L)	44.7 ± 0.2	44.7 ± 0.2	0.913	44.7 ± 0.2	45.0 ± 0.2	0.252	44.7 ± 0.2	44.4 ± 0.2	0.220
Globulin (g/L)	26.8 ± 0.2	26.6 ± 0.2	0.413	26.8 ± 0.2	26.1 ± 0.3	**0.040**	26.8 ± 0.3	27.0 ± 0.3	0.545

ALT, alanine transaminase. ALP, alkaline phosphatase. AST, aspartate transaminase. CRP, C-reactive protein. eGFR, estimated glomerular filtration rate. All values are presented as LSM ± SE. LSM: Least Squares Mean, SE: Standard Error. LSM are from repeated measures analysis of covariance with factors for visit, study group, study group by visit interaction, sex, study group by sex interaction, study group by sex by visit interaction, hospital admission in the last 30 days at baseline, baseline MUST risk, baseline age, baseline BMI, and baseline measurement. *p*-value is from the difference in LSM in study groups from the study group by sex interaction. ^ Urea to creatinine ratio was log-transformed due to skewed residuals. When the sample sizes are less than the overall stated sample sizes, the actual sample sizes are specified. The bolded values are statistically significant ($p < 0.05$).

Table S3b: Biochemical indices at day 90 and day 180 for females

**Biochemical indices**	**Overall**			**Day 90**			**Day 180**		
	**Intervention**	**Placebo**	***p*-Value**	**Intervention**	**Placebo**	***p*-Value**	**Intervention**	**Placebo**	***p*-Value**
	(*n* = 368)	(*n* = 398)		(*n* = 184)	(*n* = 204)		(*n* = 184)	(*n* = 194)	
Sodium (mmol/L)	141.1 ± 0.2	141.2 ± 0.2	0.373	141.1 ± 0.2	141.1 ± 0.2	0.863	141.1 ± 0.2	141.3 ± 0.2	0.183
Potassium (mmol/L)	4.6 ± 0.03	4.5 ± 0.03	0.050	4.6 ± 0.03	4.5 ± 0.03	0.142	4.6 ± 0.03	4.5 ± 0.03	0.088
Chloride (mmol/L)	101.8 ± 0.2	102.2 ± 0.2	**0.021**	101.6 ± 0.2	102.0 ± 0.2	0.058	101.9 ± 0.2	102.4 ± 0.2	**0.045**
Urea (mmol/L)	6.0 ± 0.1	5.3 ± 0.1	**<0.001**	6.1 ± 0.1	5.4 ± 0.1	**<0.001**	5.9 ± 0.1	5.3 ± 0.1	**<0.001**
Creatinine (μmol/L)	72.2 ± 0.8	72.9 ± 0.8	0.365	72.9 ± 0.8	73.4 ± 0.8	0.531	71.5 ± 0.9	72.4 ± 0.9	0.363
Urea to creatinine ratio ^	4.45 ± 0.02	4.31 ± 0.02	**<0.001**	4.47 ± 0.02	4.31 ± 0.02	**<0.001**	4.44 ± 0.02	4.32 ± 0.02	**<0.001**
Glucose (mmol/L)	5.5 ± 0.1	5.4 ± 0.1	0.139	5.4 ± 0.1	5.4 ± 0.1	0.406	5.6 ± 0.1	5.4 ± 0.1	0.091
eGFR (mL/min/$1.73m^2$)	79.4 ± 0.6	78.8 ± 0.6	0.325	78.8 ± 0.7	78.4 ± 0.6	0.670	80.0 ± 0.7	79.1 ± 0.7	0.200
CRP (mg/L)	4.5 ± 1.0	4.2 ± 0.9	0.815	4.6 ± 1.3	4.5 ± 1.2	0.936	4.3 ± 1.0	4.0 ± 1.0	**0.008**
	(*n* = 167)	(*n* = 181)		(*n* = 82)	(*n* = 96)		(*n* = 85)	(*n* = 85)	
Ferritin (μg/L)	191.4 ± 5.6	200.4 ± 5.6	0.129	185.8 ± 6.0	201.5 ± 6.0	**0.017**	197.1 ± 6.1	199.4 ± 6.1	0.733
Prealbumin (mg/dL)	24.6 ± 0.3	23.9 ± 0.3	**0.012**	24.9 ± 0.3	23.9 ± 0.3	**0.001**	24.3 ± 0.3	24.0 ± 0.3	0.251
Corrected calcium (mmol/L)	2.24 ± 0.01	2.23 ± 0.01	**0.040**	2.24 ± 0.01	2.23 ± 0.01	0.091	2.24 ± 0.01	2.23 ± 0.01	0.106
Vitamin B_{12} (pmol/L)	502.9 ± 10.2	420.4 ± 10.2	**<0.001**	490.5 ± 10.3	418.7 ± 10.3	**<0.001**	515.2 ± 11.3	422.2 ± 11.3	**<0.001**
	(*n* = 359)	(*n* = 387)		(*n* = 180)	(*n* = 197)		(*n* = 179)	(*n* = 190)	
Zinc (μg/L)	834.3 ± 10.6	821.3 ± 10.3	0.150	836.2 ± 11.5	820.9 ± 11.1	0.156	832.5 ± 11.7	821.7 ± 11.2	0.335
	(*n* = 238)	(*n* = 283)		(*n* = 120)	(*n* = 143)		(*n* = 118)	(*n* = 140)	
Total bilirubin (μmol/L)	10.5 ± 0.3	10.8 ± 0.3	0.229	10.4 ± 0.3	10.5 ± 0.3	0.775	10.6 ± 0.3	11.2 ± 0.3	0.075
	(*n* = 366)			(*n* = 183)			(*n* = 183)		
ALP (U/L)	63.2 ± 1.0	64.9 ± 1.0	0.114	63.7 ± 1.0	65.0 ± 1.0	0.240	62.7 ± 1.2	64.8 ± 1.2	0.124
ALT (U/L)	18.1 ± 0.9	16.0 ± 0.9	0.053	17.9 ± 0.8	16.6 ± 0.8	0.101	18.2 ± 1.4	15.5 ± 1.4	0.135
AST (U/L)	24.4 ± 0.8	23.0 ± 0.8	0.099	24.3 ± 0.8	23.4 ± 0.8	0.242	24.5 ± 1.1	22.7 ± 1.0	0.152
Total protein (g/L)	71.9 ± 0.3	71.4 ± 0.3	0.098	71.8 ± 0.3	71.1 ± 0.3	**0.022**	72.0 ± 0.3	71.8 ± 0.3	0.551
Albumin (g/L)	44.9 ± 0.2	45.0 ± 0.2	0.698	45.0 ± 0.2	44.8 ± 0.2	0.543	44.9 ± 0.2	45.2 ± 0.2	0.235
Globulin (g/L)	26.9 ± 0.2	26.4 ± 0.2	**0.016**	26.8 ± 0.2	26.2 ± 0.2	**0.019**	27.1 ± 0.2	26.6 ± 0.2	0.071

ALT, alanine transaminase. ALP, alkaline phosphatase. AST, aspartate transaminase. CRP, C-reactive protein. eGFR, estimated glomerular filtration rate. All values are presented as LSM ± SE. LSM: Least Squares Mean, SE: Standard Error. LSM are from repeated measures analysis of covariance with factors for visit, study group, study group by visit interaction, sex, study group by sex interaction, study group by sex by visit interaction, hospital admission in the last 30 days at baseline, baseline MUST risk, baseline age, baseline BMI, and baseline measurement. *p*-value is from the difference in LSM in study groups from the study group by sex interaction. ^ Urea to creatinine ratio was log-transformed due to skewed residuals. When the sample sizes are less than the overall stated sample sizes, the actual sample sizes are specified. The bolded values are statistically significant ($p < 0.05$).

Table S4a: Hematological indices at day 90 and day 180 for males

Hematological indices	Overall			Day 90			Day 180		
	**Intervention**	**Placebo**	***p*-Value**	**Intervention**	**Placebo**	***p*-Value**	**Intervention**	**Placebo**	***p*-Value**
	(*n* = 259)	(*n* = 212)		(*n* = 133)	(*n* = 109)		(*n* = 126)	(*n* = 103)	
Hemoglobin (g/dL)	13.3 ± 0.1	13.2 ± 0.1	0.286	13.4 ± 0.1	13.3 ± 0.1	0.261	13.3 ± 0.1	13.2 ± 0.1	0.423
Hematocrit (%)	40.1 ± 0.2	39.9 ± 0.2	0.380	40.3 ± 0.2	40.1 ± 0.2	0.328	39.9 ± 0.2	39.7 ± 0.2	0.544
MCV (fL)	90.4 ± 0.2	89.9 ± 0.2	**0.038**	90.3 ± 0.2	89.9 ± 0.2	0.111	90.6 ± 0.2	90.0 ± 0.2	**0.029**
MCH (pg)	30.0 ± 0.1	29.9 ± 0.1	0.109	29.9 ± 0.1	29.8 ± 0.1	0.273	30.1 ± 0.1	29.9 ± 0.1	0.078
MCHC (g/dL)	33.3 ± 0.1	33.2 ± 0.1	0.549	33.2 ± 0.1	33.2 ± 0.1	0.676	33.3 ± 0.1	33.2 ± 0.1	0.539
RDW (%)	13.24 ± 0.06	13.35 ± 0.06	0.138	13.27 ± 0.07	13.30 ± 0.07	0.755	13.21 ± 0.07	13.40 ± 0.07	**0.025**
Platelet count ($10^3/\mu L$)	208.2 ± 3.6	215.8 ± 3.8	0.084	207.6 ± 3.9	216.2 ± 4.1	0.076	208.7 ± 4.0	215.4 ± 4.3	0.191
MPV (fL)	10.0 ± 0.04	10.0 ± 0.05	0.176	10.1 ± 0.1	10.0 ± 0.1	0.074	10.0 ± 0.1	10.0 ± 0.1	0.544
	(*n* = 254)	(*n* = 205)		(*n* = 130)	(*n* = 106)		(*n* = 124)	(*n* = 99)	
WBC count ($10^3/\mu L$)	5.9 ± 0.1	5.8 ± 0.1	0.794	6.0 ± 0.2	5.8 ± 0.2	0.194	5.7 ± 0.1	5.9 ± 0.1	0.251
Neutrophils (absolute) ($10^3/\mu L$)	3.5 ± 0.1	3.5 ± 0.1	0.857	3.6 ± 0.1	3.4 ± 0.1	0.266	3.4 ± 0.1	3.5 ± 0.1	0.234
Lymphocytes (absolute) ($10^3/\mu L$)	1.7 ± 0.03	1.7 ± 0.03	0.800	1.7 ± 0.04	1.7 ± 0.04	0.823	1.7 ± 0.04	1.7 ± 0.04	0.529
Monocytes (absolute) ($10^3/\mu L$)	0.51 ± 0.01	0.49 ± 0.01	0.155	0.53 ± 0.01	0.48 ± 0.01	**0.005**	0.49 ± 0.01	0.50 ± 0.01	0.690
Eosinophils (absolute) ($10^3/\mu L$)	0.21 ± 0.01	0.20 ± 0.01	0.586	0.22 ± 0.02	0.21 ± 0.02	0.900	0.21 ± 0.01	0.19 ± 0.01	0.364
Basophils (absolute) ($10^3/\mu L$)	0.04 ± 0.004	0.04 ± 0.004	0.857	0.04 ± 0.004	0.04 ± 0.004	0.623	0.04 ± 0.004	0.04 ± 0.004	0.859
Neutrophils (%)	57.8 ± 0.6	58.0 ± 0.6	0.863	58.1 ± 0.7	57.5 ± 0.7	0.556	57.6 ± 0.7	58.4 ± 0.7	0.373
Lymphocytes (%)	28.9 ± 0.5	29.3 ± 0.6	0.508	28.7 ± 0.6	29.6 ± 0.6	0.231	29.2 ± 0.6	29.1 ± 0.6	0.950
Monocytes (%)	8.9 ± 0.1	8.6 ± 0.1	0.106	8.9 ± 0.2	8.6 ± 0.2	0.069	8.8 ± 0.2	8.6 ± 0.2	0.310
Eosinophils (%)	3.6 ± 0.2	3.3 ± 0.2	0.233	3.5 ± 0.2	3.5 ± 0.2	0.949	3.6 ± 0.2	3.1 ± 0.2	**0.040**
Basophils (%)	0.76 ± 0.03	0.76 ± 0.03	0.905	0.79 ± 0.03	0.77 ± 0.03	0.646	0.74 ± 0.03	0.75 ± 0.03	0.782
RBC count ($10^6/\mu L$)	4.5 ± 0.02	4.5 ± 0.03	0.667	4.5 ± 0.02	4.5 ± 0.03	0.836	4.4 ± 0.03	4.5 ± 0.03	0.379
Reticulocytes (absolute) ($10^3/\mu L$)	62.2 ± 1.1	58.7 ± 1.1	**0.008**	61.8 ± 1.2	58.2 ± 1.2	**0.014**	62.5 ± 1.2	59.1 ± 1.3	**0.006**
Reticulocytes (%)	1.4 ± 0.03	1.3 ± 0.03	**0.007**	1.4 ± 0.03	1.3 ± 0.03	**0.024**	1.4 ± 0.03	1.3 ± 0.03	**0.013**

MCH, mean corpuscular hemoglobin. MCHC, mean corpuscular hemoglobin concentration. MCV, mean corpuscular volume. MPV, mean platelet volume. RBC, red blood cell. RDW, red cell distribution width. WBC, white blood cell. All values are presented as LSM ± SE, unless otherwise stated. LSM: Least Squares Mean, SE: Standard Error. LSM are from repeated measures analysis of covariance with factors for visit, study group, study group by visit interaction, sex, study group by sex interaction, study group by sex by visit interaction, hospital admission in the last 30 days at baseline, baseline MUST risk, baseline age, baseline BMI, and baseline measurement. *p*-value is from the difference in LSM in study groups from the study group by sex interaction. When the sample sizes are less than the overall stated sample sizes, the actual sample sizes are specified. The bolded values are statistically significant ($p < 0.05$).

Table S4b: Hematological indices at day 90 and day 180 for females

Hematological indices	Overall			Day 90			Day 180		
	**Intervention**	**Placebo**	***p*-Value**	**Intervention**	**Placebo**	***p*-Value**	**Intervention**	**Placebo**	***p*-Value**
	(*n* = 365)	(*n* = 399)		(*n* = 183)	(*n* = 205)		(*n* = 182)	(*n* = 194)	
Hemoglobin (g/dL)	13.1 ± 0.1	13.1 ± 0.1	0.312	13.2 ± 0.1	13.1 ± 0.1	0.147	13.1 ± 0.1	13.0 ± 0.1	0.683
Hematocrit (%)	39.7 ± 0.2	39.4 ± 0.2	0.190	39.8 ± 0.2	39.4 ± 0.2	0.065	39.5 ± 0.2	39.4 ± 0.2	0.574
MCV (fL)	90.1 ± 0.2	90.0 ± 0.2	0.789	90.1 ± 0.2	90.0 ± 0.2	0.644	90.1 ± 0.2	90.0 ± 0.2	0.945
MCH (pg)	29.9 ± 0.1	29.9 ± 0.1	0.792	29.9 ± 0.1	29.9 ± 0.1	0.745	29.9 ± 0.1	29.9 ± 0.1	0.855
MCHC (g/dL)	33.1 ± 0.1	33.2 ± 0.1	0.374	33.1 ± 0.1	33.2 ± 0.1	0.282	33.1 ± 0.1	33.1 ± 0.08	0.589
RDW (%)	13.25 ± 0.06	13.21 ± 0.06	0.427	13.21 ± 0.06	13.18 ± 0.06	0.700	13.30 ± 0.06	13.23 ± 0.06	0.323
Platelet count ($10^3/\mu L$)	219.6 ± 3.3	219.4 ± 3.3	0.940	220.0 ± 3.5	219.3 ± 3.5	0.854	219.2 ± 3.6	219.4 ± 3.6	0.963
MPV (fL)	10.0 ± 0.04	9.9 ± 0.04	**0.002**	10.0 ± 0.04	9.9 ± 0.04	**0.001**	10.0 ± 0.04	9.9 ± 0.04	**0.044**
	(*n* = 356)	(*n* = 398)		(*n* = 179)	(*n* = 204)		(*n* = 177)		
WBC count ($10^3/\mu L$)	5.7 ± 0.1	5.6 ± 0.1	0.718	5.7 ± 0.1	5.7 ± 0.1	0.999	5.6 ± 0.1	5.5 ± 0.1	0.497
		(*n* = 398)			(*n* = 204)				
Neutrophils (absolute) ($10^3/\mu L$)	3.3 ± 0.1	3.3 ± 0.1	0.609	3.4 ± 0.1	3.4 ± 0.1	0.902	3.3 ± 0.1	3.2 ± 0.1	0.403
Lymphocytes (absolute) ($10^3/\mu L$)	1.6 ± 0.03	1.7 ± 0.03	0.175	1.6 ± 0.03	1.7 ± 0.03	0.067	1.6 ± 0.03	1.6 ± 0.03	0.573
Monocytes (absolute) ($10^3/\mu L$)	0.47 ± 0.01	0.46 ± 0.01	0.370	0.47 ± 0.01	0.46 ± 0.01	0.490	0.47 ± 0.01	0.46 ± 0.01	0.416
Eosinophils (absolute) ($10^3/\mu L$)	0.21 ± 0.01	0.19 ± 0.01	0.078	0.22 ± 0.01	0.19 ± 0.01	0.064	0.21 ± 0.01	0.19 ± 0.01	0.288
Basophils (absolute) ($10^3/\mu L$)	0.04 ± 0.003	0.04 ± 0.003	0.733	0.04 ± 0.004	0.04 ± 0.004	0.874	0.04 ± 0.004	0.04 ± 0.004	0.689
Neutrophils (%)	58.5 ± 0.6	57.6 ± 0.6	0.135	58.8 ± 0.6	57.9 ± 0.6	0.175	58.1 ± 0.6	57.3 ± 0.6	0.227
Lymphocytes (%)	28.9 ± 0.5	30.1 ± 0.5	**0.015**	28.5 ± 0.5	30.0 ± 0.5	**0.011**	29.3 ± 0.5	30.2 ± 0.5	0.102
Monocytes (%)	8.4 ± 0.1	8.3 ± 0.1	0.335	8.4 ± 0.1	8.2 ± 0.1	0.258	8.4 ± 0.1	8.3 ± 0.1	0.577
Eosinophils (%)	3.4 ± 0.2	3.2 ± 0.2	0.254	3.5 ± 0.2	3.2 ± 0.2	0.133	3.4 ± 0.2	3.3 ± 0.2	0.667
Basophils (%)	0.78 ± 0.02	0.80 ± 0.02	0.659	0.78 ± 0.03	0.78 ± 0.03	0.951	0.79 ± 0.02	0.81 ± 0.03	0.465
RBC count ($10^6/\mu L$)	4.5 ± 0.02	4.4 ± 0.02	0.153	4.5 ± 0.02	4.4 ± 0.02	0.070	4.5 ± 0.03	4.4 ± 0.03	0.398
Reticulocytes (absolute) ($10^3/\mu L$)	61.8 ± 1.0	57.7 ± 1.0	**<0.001**	61.1 ± 1.1	56.5 ± 1.0	**<0.001**	62.6 ± 1.1	58.9 ± 1.1	**0.002**
Reticulocytes (%)	1.4 ± 0.02	1.3 ± 0.02	**0.001**	1.4 ± 0.03	1.3 ± 0.03	**<0.001**	1.4 ± 0.03	1.3 ± 0.03	**0.008**

MCH, mean corpuscular hemoglobin. MCHC, mean corpuscular hemoglobin concentration. MCV, mean corpuscular volume. MPV, mean platelet volume. RBC, red blood cell. RDW, red cell distribution width. WBC, white blood cell. All values are presented as LSM ± SE, unless otherwise stated. LSM: Least Squares Mean, SE: Standard Error. LSM are from repeated measures analysis of covariance with factors for visit, study group, study group by visit interaction, sex, study group by sex interaction, study group by sex by visit interaction, hospital admission in the last 30 days at baseline, baseline MUST risk, baseline age, baseline BMI, and baseline measurement. *p*-value is from the difference in LSM in study groups from the study group by sex interaction. When the sample sizes are less than the overall stated sample sizes, the actual sample sizes are specified. The bolded values are statistically significant ($p < 0.05$).
